# Wolff-Parkinson-White Syndrome in Third Trimester of Pregnancy

**DOI:** 10.7759/cureus.22731

**Published:** 2022-03-01

**Authors:** Luis F Lemus, Ingrid Pereira, Roberto Valdivieso

**Affiliations:** 1 Medical School, Universidad Dr José Matias Delgado, San Salvador, SLV; 2 Obstetrics and Gynecology, Hospital Nacional San Rafael, Santa Tecla, SLV

**Keywords:** cardioversion, electrocardiogram, pregnancy, supraventricular tachycardia, wolff-parkinson-white syndrome

## Abstract

Wolff-Parkinson-White (WPW) syndrome is rare and is characterized by an accessory pathway that predisposes patients to tachyarrhythmias and sudden cardiac death. Early recognition is important and should be evaluated by a multidisciplinary team for adequate management and treatment. We present a pregnant woman that presented to the emergency department and discuss her diagnosis, treatment, and outcome.

## Introduction

As maternal morbidity and mortality continue to rise, arrhythmias have emerged as the most common cardiovascular complication of pregnancy in women with and without structural heart disease. Appropriate maternal diagnosis and management are important to optimize maternal and fetal outcomes. Supraventricular tachycardia (SVT) is considered the most frequent sustained arrhythmia in pregnancy [1]. Wolff-Parkinson-White (WPW) syndrome is a rare congenital cardiac pre-excitation syndrome that may result in symptomatic and life-threatening arrhythmias [2]. 

Here we present a case report that highlights a pregnant woman with supraventricular tachycardia in the emergency department. Physical examination, clinical course, ECG features, and treatment are discussed.

## Case presentation

A 23-year-old woman at 35 weeks + two days of gestation by amenorrhea (G1 P0 L0) presented to the emergency department with chest pain, palpitations, and dizziness. The patient had no vaginal bleeding or vaginal hydrorrhea. On physical examination, the patient had a heart rate of 215 beats per minute and respiratory rate of 26 breaths per minute but was hemodynamically stable (122/60 mmHg). The cardiovascular and pulmonary examination was otherwise unremarkable. Her first and second trimester was uneventful; the patient has a history of sinus tachycardia since her childhood managed with oral propranolol during pregnancy. She had no other relevant medical history or family history of cardiac history. Blood count, renal function test, electrolytes, bilirubin, coagulation test were performed which were normal. The liver function test showed a mild elevation of AST(59 U/L) and normal ALT.

**Figure 1:**
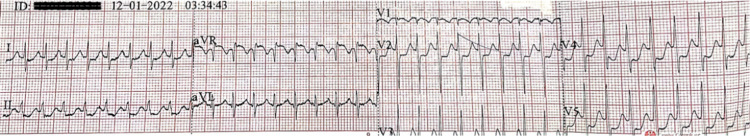
ECG on admission showing narrow complex tachycardia with hidden P waves.

An electrocardiogram (ECG) was performed during the episode which showed narrow complex tachycardia findings of supraventricular tachycardia (Figure 1). 

**Figure 2:**
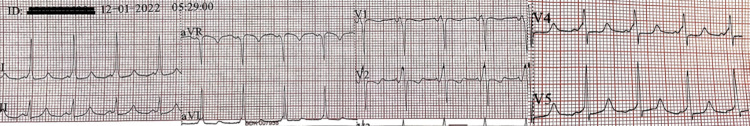
ECG following verapamil showing sinus rhythm with short PR interval, delta wave and prolonged QRS duration.

Vagal maneuvers such as carotid massage and Valsalva maneuvers failed to correct the pulse. Due to the lack of adenosine in our institution, 5 mg of intravenous verapamil was given in bolus. The patient regained normal heart rate after treatment and the ECG showed findings of Wolff-Parkinson-White (WPW) syndrome (Figure 2).

The fetus was in a longitudinal position, with good vital signs; the cardiotocograph was reactive with good variability and no uterine contractions. Two more episodes of supraventricular tachycardia occurred but the patient remained stable with the same pharmacological treatment mentioned above. The patient was subsequently referred to a local tertiary center where she underwent a cesarean delivery and her pharmacological treatment was changed to propafenone (150 mg PO q8hr). Two weeks later the patient presented to the emergency department with palpitations and a heart rate of 230 bpm with ECG showing supraventricular tachycardia unresponsive to vagal maneuvers and pharmacological treatment with a blood pressure of 46/24 mmHg, therefore, cardioversion at 150 joules was performed. After the episode, the patient was transferred to a tertiary center for the management of her cardiac condition.

## Discussion

WPW syndrome was first described in 1930 and is characterized by one or more atrioventricular accessory pathways that predispose patients to recurrent paroxysmal episodes of supraventricular tachycardia [3]. The general prevalence of WPW has been estimated between 0.1 to 0.3% of the population and the incidence of patients that progress to arrhythmia is around 1% to 2% per year [4]. This syndrome can be associated with a malignant arrhythmia resulting in sudden death. Symptomatic patients have an estimated risk reported to be approximately 0.25% per year or 3% to 4% over a lifetime [5].

Patients with WPW who develop tachyarrhythmia might experience symptoms such as palpitations, chest pain, dyspnea, dizziness, syncope, and/or sudden death. The hallmark electrocardiographic finding of WPW pattern or preexcitation consists of a short PR interval and prolonged QRS with an initial slurring upstroke ("delta" wave) in the presence of sinus rhythm [2].

The 2015 ACC/AHA/HRS SVT guidelines recommend acute conversion of SVT, vagal maneuvers including Valsalva, and carotid sinus massage for acute conversion which should be performed quickly as a first-line intervention to terminate SVT. When vagal maneuvers fail to terminate SVT, adenosine is the first-line pharmacological option for pregnant patients. Intravenous verapamil is an effective treatment; however, there is a higher risk of maternal hypotension [6-7].

Synchronized cardioversion is recommended for acute treatment in pregnant patients with hemodynamically unstable SVT when pharmacological therapy is ineffective or contraindicated [6].

Catheter ablation is recommended in pregnant patients with highly symptomatic who have failed multiple drug efforts. If a catheter ablation procedure is required during pregnancy, radiation-reduction technologies should be used, and the procedure should be avoided in the first trimester when the teratogenic risk is greater [8-9].

## Conclusions

Wolff-Parkinson-White syndrome is a rare condition and diagnosis might be missed. Identification of this disease is important to prevent further arrhythmias and sudden cardiac death. Arrhythmias can be life-threatening to both the fetus and the mother, so close monitoring should be done. Management of this condition should be done by a multidisciplinary team.
